# Foreign Summary

**Published:** 1840-03-07

**Authors:** 


### FOREIGN SUMMARY.

*Treatment of Dysmenorrhœa with Mercurial Alteratives.* By Robert Dunlop, Surgeon.—Having been repeatedly foiled in my attempts to subdue this painful disease, after well-directed medical treatment had been continued for a due space of time, I was led to make trial of mercurial alteratives, from the well known fact of their efficacy in relieving painful affections of many other organs, more especially those affections of a subacute inflammatory type. The result has been favourable, so far as my own experience has gone, and I subjoin the two following cases as an example:—

*Case* 1.—M. Irvine, aged twenty-eight, of a rather open habit of body, has been married about five years; never has been pregnant; catamenia she states to have been regular since the age of fifteen or sixteen, at which period she first began to menstruate; no remarkable deviation has ever taken place, either as regards the quantity or quality of the discharge; her periods have been passed with little inconvenience until about two years ago, when the painful symptoms first began to manifest themselves; each period since has been more or less attended with excruciating suffering, which was of short duration in the early stage of the complaint, and relieved by the menstrual discharge, but subsequently the pain has continued throughout the whole period, enduring a day or two after the discharge had totally ceased. Her present symptoms are: bowels costive; countenance sallow, with a somewhat haggard appearance; catamenia regular; no tenderness upon pressure on the sacrum; paroxysms of pain lasting from six to eight days, and so severe as to compel her frequently to assume the horizontal posture; she remains tolerably free from pain in the intervals, but describes her present existence to be almost insupportable, from the unerring certainty of having to undergo at each period the same acute suffering.

*April* 16, 1838.—I ordered the warm hip-bath, and flannels wrung out of hot water to the pubes, together with opiates, during the attack, with great relief; in the interval, her general health was attended to, and her bowels kept sufficiently open; she took for some time preparations of iron, a course of the ioduret of potassium, and, lastly, guaiacum three or four days previous to the expected attack; she took, likewise, the ext. of hyoscyamus, with considerable doses of camphor and the semicupium at bedtime; the result was a temporary relief only, and a gradual return of all her sufferings; she had now passed over six periods, with little prospect of procuring any permanent relief, when I ordered her the following pills, to be commenced at least six or eight days previous to the expected attack:—

℞.—Blue Pill, one drachm;
Opium, 6 grains;

Well compounded and divided into sixteen pills. Take one, night and morning, until twelve are taken. Afterwards, one every night.

℞.—Infusion of columba root, 7 ounces;
Tincture of gentian, 1 ounce;
Carbonate of soda, 1 drachm.

Make a mixture. Take two table-spoonfuls two or three times a day.

She passed over the usual period with scarcely a vestige of pain, and expressed great relief; the discharge she thought was slightly increased in quantity; she took no more medicine, and passed over the next period with a slight pain only; fearing a relapse I prescribed the following pills to be given eight days before her expected period:—

℞.—Mercury, with chalk;
Dover's powder; of each 2 scruples;
Extract of black hyoscyamus, half a drachm.

Make twenty-four pills. Take two every night at bed time.

The result has been a complete relief from her sufferings, and great improvement in her general health.

*Case* 2, *October*, 1838.—A. Newton, aged thirty, married, of a full and lax habit of body; has had one child, about four years ago; no remarkable deviation has ever occurred in menstruation, until about four months ago, when painful menstruation first began; she complains of an almost constant aching pain in the back, loins, and thighs, symptoms which are greatly aggravated at each period: menses much more profuse than usual, and continue longer than natural; sacrum slightly tender on pressure; tongue furred; pulse eighty.

Leeches were first applied to the sacrum in this case, together with cooling aperients and low diet; in the course of time various remedies were had recourse to, together with sedatives when required, but no complete or lasting relief was obtained. I again ordered mercurial alteratives much as in the former case, with complete success; not only were the painful symptoms speedily subdued, but a simultaneous diminution of the catamenia took place.

*Remarks.*—With reference to these cases we have to observe, that we are not acquainted with any writer who has sufficiently insisted upon the employment of mercurial alteratives in this disease; and, if we mistake not, there exists a general dislike to employing mercurials as remedial agents in dysmenorrhœa, partly from a dread of the baneful influence sometimes observed to follow the use of this mineral, and partly also from the female possessing a constitution generally thought less suited to bear the influence of this remedy, but as yet we have never witnessed any hurtful effects to follow its use as here recommended. There are no doubt other forms of mercury, as being better suited to certain constitutions, which must be left to the judgment of the practitioner to adopt, but as yet we have never found it necessary to have recourse to any other forms than those already specified, which have always been successful in our hands. But whilst we thus strenuously advocate the use of mercurial alteratives, we are far from inferring that every cause of dysmenorrhœa should be treated by this remedy; we, however, wish to be distinctly understood that every case of obstinacy, after having resisted general treatment, and where there is no particular organic disease forbidding the use of mercury, should be treated by this remedy as a very certain means of subduing this painful affection.

*Lancet.*

---

*Cases of Club-foot successfully treated by operation.*

*Case* 1.—Mad. Bidaine, 37 years of age, has been affected with the *pes equinus* since she was five years of age. It was the result of an abscess in the ham and calf of the leg, which had left behind a permanent contraction of the tendo-Achillis. The consequence of this was, that the patient had never been able to put the foot fairly down upon the ground, but always walked on her toes. The size of the affected limb was much smaller than its fellow.

Notwithstanding the number of years that the deformity had existed, the patient was so anxious that an attempt be made to remedy her deformity, that Dr. Lenger determined to perform the operation of dividing the tendo-Achillis. On the fifth day afterwards, the adhesive bandage was removed, and the wound was then found to be cicatrised. A slight depression was perceptible between the divided ends of the tendon. The patient experienced a dragging pain in the leg, extending upwards along the thigh; the foot was somewhat swollen, and had, greatly to the surprise of the patient, already recovered in a considerable degree its natural position, forming now nearly a right angle with the leg. We should mention that a simple *bottine à tuteur* was worn by the patient. Before the end of the second week she could bend the foot at the ankle joint; and daily this movement became more and more easy. Whenever the boot was removed, the foot was immediately drawn into its faulty position, the force of the powerful extensor muscles being still much greater than that of the flexors.

It was indeed doubtful whether, at the patient's time of life, this predominance might ever be overcome. After the lapse of several months, however, she was able to walk about with an ordinary boot; and although she still limped a little in consequence of a slight shortening of the affected limb, the foot had entirely regained its normal position.

The case may be regarded as altogether one of the most satisfactory proofs of what may be effected by surgical assistance in one of the varieties of club-foot.

*Case* 2.—Marie Lacomte, 11 years of age, was affected with congenital varus (the foot turned inwards) of the left foot. The tendo-Achillis was divided at about two inches above the heel. The *bottine à tuteur* was put on immediately after the operation. Every thing went on favourably, and the deformed foot speedily acquired a permanent healthy direction. The cure was complete at the end of a month. Four months afterwards it was scarcely possible to perceive the slightest difference between the two feet in walking; for six weeks she had discontinued the use of the *bottine*, and had worn a common shoe.

*Case* 3.—A boy, 7 years old, was born with *varus* of both feet. Every sort of bandage and apparatus had been tried to rectify the deformity; but in vain. Indeed, these attempts seem to have rather increased than diminished the tendency of the feet to turn inwards. The legs, especially the calves, were unusually small, and the feet appeared to be out of joint (*deboités*,) the tibio-tarsal spaces being quite straight. On the 17th of July, the tendo-Achillis of each limb was divided at about an inch and a half above their insertion into the heel. The *bottines* were immediately put on. These he continued to wear for several months. Ultimately a perfect cure was obtained.

*Case* 4.—A young man, 17 pears of age, was affected with congenital varus of both feet. The deformity was very great, in consequence of the large bosses or tumours which the indurated skin had formed over the insteps. He had the greatest difficulty in walking any distance, and his situation was altogether miserable. In other respects, this patient was healthy and robust for his age.

Both tendons were divided at between two and three inches above the distal insertion; and the *bottines* were put on immediately afterwards. Large eschars formed over the insteps; but these gradually separated, and the ulcers healed up, although the *bottines* were kept constantly applied. In six weeks he could walk about alone; and in two months more he began to wear a pair of common boots, to which a semicircular steel strap was affixed. The cure may be considered as complete.

Dr. Lenger alludes to several other cases—in all sixteen—in which he has performed the operation of dividing the tendo-Achillis. In every one of these, he has succeeded in removing the deformity; and in none has any disagreeable accident occurred.

In very young children, the deformity may certainly be often cured without any operation, and merely by the use of an appropriate bandage and of a boot, so contrived as to bring and retain the foot in a normal position.—*Med. Chirurgical Review, from Bulletin Medical Belge.*

